# Preoperative evaluation profile of patients undergoing arterial vascular surgery in a tertiary hospital

**DOI:** 10.1016/j.clinsp.2024.100445

**Published:** 2024-07-25

**Authors:** Arthur Souza Magnani, Leandro Teixeira de Castro, Isabela Cristina Kirnew Abud Manta, Viviane Galli Dib, Luiz Otávio Vittorelli, Felipe Soares Oliveira Portela, Nelson Wolosker, Marcelo Passos Teivelis

**Affiliations:** a Faculdade Israelita de Ciências da Saúde Albert Einstein, Hospital Israelita Albert Einstein, São Paulo, SP, Brazil; b Hospital Municipal da Vila Santa Catarina Dr. Gilson de Cássia Marques de Carvalho; Hospital Israelita Albert Einstein, São Paulo, SP, Brazil; c Faculdade de Medicina da Universidade de São Paulo (FMUSP), São Paulo, SP, Brazil

**Keywords:** Preoperative evaluation, Major adverse cardiac events, Non-cardiac vascular surgery

## Abstract

**Background:**

Patients with peripheral arterial disease have an increased risk of developing cardiovascular complications in the postoperative period of arterial surgeries known as Major Adverse Cardiac Events (MACE), which includes acute myocardial infarction, heart failure, malignant arrhythmias, and stroke. The preoperative evaluation aims to reduce mortality and the risk of MACE. However, there is no standardized approach to performing them. The aim of this study was to compare the preoperative evaluation conducted by general practitioners with those performed by cardiologists.

**Methods:**

This is a retrospective analysis of medical records of patients who underwent elective arterial surgeries from January 2016 to December 2020 at a tertiary hospital in São Paulo, Brazil. The authors compared the preoperative evaluation of these patients according to the initial evaluator (general practitioners vs. cardiologists), assessing patients' clinical factors, mortality, postoperative MACE incidence, rate of requested non-invasive stratification tests, length of hospital stay, among others.

**Results:**

281 patients were evaluated: 169 assessed by cardiologists and 112 by general practitioners. Cardiologists requested more non-invasive stratification tests (40.8%) compared to general practitioners (9%) (*p* < 0.001), with no impact on mortality (8.8% versus 10.7%; *p* = 0.609) and postoperative MACE incidence (10.6% versus 6.2%; *p =* 0.209). The total length of hospital stay was longer in the cardiologist group (17.27 versus 11.79 days; *p <* 0.001).

**Conclusion:**

The increased request for exams didn't have a significant impact on mortality and postoperative MACE incidence, but prolonged the total length of hospital stay. Health managers should consider these findings and ensure appropriate utilization of human and financial resources.

## Highlights

• Cardiovascular complications are common in the postoperative period of peripheral arterial vascular surgeries.
• There is not a unique and standardized way to conduct preoperative evaluations in arterial vascular surgery patients.
• In the studied hospital, in different time periods, evaluations were performed by either cardiologists or hospitalists.
• Increased requests for exams had no impact on postoperative mortality or complications in this study.
• Health managers should ensure appropriate utilization of human and financial resources for comparable outcomes.

## Introduction

Patients proposed for arterial vascular surgeries have a high risk of developing cardiovascular complications in the postoperative period.^1^ These complications, known as Major Adverse Cardiovascular Events (MACE), are commonly defined in the literature as Acute Myocardial Infarction (AMI), Decompensated Heart Failure (DHF), stroke, or malignant arrhythmias (unstable ventricular or supraventricular arrhythmias).^1^^,^^2^

MACE is highly common in arteriopathies patients, with its incidence ranging from 5% to 15% in the postoperative period of non-cardiac vascular surgeries.^1^^,^^2^ The incidence of AMI ranges from 0.3% to 36%, may not present with typical symptoms (such as chest pain or dyspnea)^3^ and other patients may also develop acute Myocardial Injury after Non-cardiac Surgery (MINS).^4^ Besides specific characteristics of the patients, pathophysiology similarities with atherosclerotic disease, its systemic nature, and factors related to the arterial vascular surgery itself, such as hemodynamic instability, bleeding, clamping of major vessels, thromboembolic and reperfusion phenomena also contribute to a higher incidence of MACE.^1^^,^^2^

Clinical scales are often used to identify patients with high cardiac risk and guide the performance of non-invasive stratification tests (such as stress echocardiography, myocardial scintigraphy, or Coronary Computed Tomography Angiography [CCTA]). The evaluator can then recommend a specific therapy aiming at perioperative pharmacological protection using beta-blockers, antiplatelet agents, statins, and other agents.^5^^,^^6^ Additionally, invasive stratification through coronary angiography and, depending on the case, myocardial revascularization may be indicated.^7^^,^^8^ Many specialties can perform clinical assessment, such as anesthesiologists, cardiologists, primary care physicians, and hospitalists. Studies have already demonstrated the importance of hospitalists for preoperative assessment, reducing length of hospital stay, postoperative complications, and mortality when compared to a non-standardized preoperative assessment by other specialties.^9^^,^^10^ However, there is no standardized or universal evaluation strategy used worldwide, which leads to heterogeneity in assessments and difficulties in comparing the efficiency of adopted approaches.^11^

The aim of this study was to compare the preoperative evaluation of arterial vascular surgeries conducted by hospitalists with those performed by cardiologists at a tertiary hospital with approximately 240 beds in São Paulo, Brazil. The authors analyzed the differences in preoperative risk stratification, request for cardiac stratification tests (invasive and non-invasive tests), the interval between the initial preoperative evaluation and surgery, the approaches adopted in each case, and the impact that all these variables had on postoperative mortality and incidence of MACE.

## Methods

This is a retrospective study that analyzed the medical records of patients undergoing arterial vascular surgery from January 2016 to December 2020 at a tertiary hospital with approximately 240 beds specializing in organ transplant and oncology treatment in São Paulo, Brazil. The project was submitted to and approved by the Research Ethics Committee of the Albert Einstein Hospital Jewish Charitable Society (CAAE number 38597520.4.0000.0071).

In this hospital, from 2016 to 2018 all cardiac risk evaluations were conducted by cardiologists. However, due to historical and administrative reasons, in 2018 internal medicine physicians began performing these evaluations, with cardiologists assessing specific cases when necessary. During this period, there were no significant changes in postoperative protocols and postoperative intensive care. The surgical volume also remained relatively the same. As for the surgical team, there was an annual turnover of resident doctors. However, there was no change in the attending surgeon's staff which may have left this permanent team of doctors more experienced at the end of 5 years of study. This enabled us to compare the preoperative evaluation of patients with similar clinical-demographic characteristics within the same hospital, separating them into two groups according to the initial evaluator (internal medicine physicians/hospitalists or cardiologists).

The authors analyzed the medical records of patients who underwent arterial vascular surgery during the study period and divided them into two groups according to the type of physician who conducted the initial preoperative evaluation: hospitalists or cardiologists.

Patients initially evaluated by a hospitalist and subsequently referred for cardiology evaluation were included in the hospitalist group, whereas patients first evaluated by a cardiologist (without prior evaluation by a hospitalist) were included in the cardiologist group. It should be noted that there was a physician in the team of hospitalists who, in addition to their general practice training, was also a cardiologist. To avoid any potential biases related to the preoperative evaluation by this professional (who worked as a hospitalist in the hospital but had a specialization in cardiology), the authors chose to exclude from the study all patients evaluated by this specific physician.

The variables analyzed were: age; sex; type of surgery performed (based on topography: aorta, carotid, lower extremity; and on technique: open or endovascular); the initial evaluator (hospitalist vs. cardiologist); number of non-invasive cardiac stratification tests performed and type of test performed (pharmacological stress echocardiogram, CCTA, or myocardial perfusion scintigraphy); number of invasive cardiac stratification tests performed with or without myocardial revascularization (percutaneous) or open myocardial revascularization; and patient risk factors such as prior stroke, Systemic Arterial Hypertension (SAH), chronic kidney disease (defined as serum creatinine level above 1.5 mg/dL), Diabetes Mellitus (DM), Heart Failure (HF), smoking, former smoking, history of myocardial revascularization, troponin levels, Brain Natriuretic Peptide (BNP) levels, and preoperative C-Reactive Protein (CRP) levels (when available).

The authors have also separated the assessments between outpatient versus inward first preoperative evaluations and the surgery performed as elective versus time-sensitive. The authors considered all limbs revascularization and all carotids operated on within 15 days after stroke as time-sensitive surgeries. All aortic aneurysm surgeries and carotids operated more than 15 days after stroke were considered elective surgeries. Other surgeries were classified according to the initial evaluator's judgment. Urgent and emergent surgeries were excluded.

The following outcomes were assessed: in-hospital mortality, length of hospital stay, Intensive Care Unit (ICU) length of stay, post-surgery hospitalization days, and occurrence of MACE in the postoperative period (until hospital discharge). In the present study, MACE was defined as decompensated HF, Acute Myocardial Infarction (AMI), stroke and ventricular arrhythmias, or any other arrhythmias associated with hemodynamic instability. The authors defined MINS as a 50% elevation of troponin levels compared to the baseline (presumably due to myocardial ischemia), without the development of anginal symptoms or electrocardiographic changes. This definition was an adaptation of the Vision Study.^12^ The diagnoses of HF, AMI, and MINS were established by the attending medical team in the postoperative period.

The authors also analyzed three time-lags, in days: between the initial preoperative evaluation and the day of surgery, between the initial evaluation and cardiac catheterization, and between the initial evaluation and myocardial revascularization (open or percutaneous).

### Inclusion criterion

‒ Patients who underwent preoperative evaluation at the Referral Hospital, both as an outpatient and during hospitalization.

### Exclusion criteria

– Patients who underwent two vascular surgeries in different sites during the same hospitalization (e.g., carotid surgery on one day and lower limb revascularization on another). Reoperations (due to complications from the first surgery) were not excluded, as they represent significant events of interest for the study;

– Patients who underwent surgery in conjunction with another specialty;

– Emergency or urgent surgeries;

– Patients under 18 years of age;

– Patients were evaluated by a physician who was part of the hospitalists’ team at the hospital but also specialized in cardiology (*n* = 13).

### Statistical analysis

Categorical variables were first described for the total population using absolute frequencies and relative frequencies (percentages), and then for the two groups. Parametric quantitative variables were first described for the total population as mean, standard deviation, minimum, and maximum values, and then for the two groups. Nonparametric quantitative variables were first described as mean, median, interquartile range, minimum and maximum values, and then for the two groups.

Quantitative variables were classified as parametric or nonparametric according to what had already been described about the characteristic of the variable in the literature. Therefore, normality tests were not applied to the variables studied. Age was classified as parametric variable; while baseline creatinine levels, preoperative BNP levels, preoperative CRP levels, preoperative troponin levels, total length of hospital stay (days), length of ICU stay (days), and postoperative length of hospital stay (days) were classified as nonparametric variables.

The outcomes of mortality and cardiovascular morbidity were evaluated according to the variables considered, particularly the initial evaluator (hospitalist vs. cardiologist). Associations between categorical variables and each outcome were assessed using association tests such as the Chi-Square test, Fisher's exact test, and likelihood ratio test. Quantitative characteristics were compared according to the outcomes using t-Student tests for parametric variables or Mann-Whitney tests for non-parametric variables. All analyses were performed using IBM SPSS Statistics for Windows version 22.0 software (IBM Corp., Armonk, NY, USA), and the significance level was set at 5%.

## Results

From January 2016 to December 2020, Hospitalists have performed 112 preoperative evaluations (39.8%) and cardiologists have performed 169 (60.2%), resulting in 281 evaluations in total. 131 surgeries were performed from January 2016 to December 2017 and 181 were performed from January 2018 to December 2020 totalizing 312 surgeries. As some patients developed complications that required further surgeries, the number of surgeries was greater than the number of preoperative evaluations. In these cases, the preoperative assessment referred to the first surgery, since it was not possible to predict which patients would undergo other surgeries due to complications.

The population was predominantly made up of male patients (*n =* 193/68.7%), with 88 female patients (31.3%). The mean age of the total population was 66.1 years, being 66.07 years in the general practitioners’ group and 66.12 years in the cardiologists’ group (*p =* 0.971). The clinical-demographic profile of the patients was similar between both groups as shown in Table 1 (for additional clinical-demographic data, please refer to Supplementary Tables 1, 2, and 3 in Supplementary_File). Of all patients, 27 evolved to death (9.6%), 25 patients evolved with MACE (8.8%) and 45 patients (16%) evolved with MINS (Table 3, Table 4). There was no statistical difference regarding mortality, incidence of MACE, or MINS between initial evaluators.

**Table 1:** Type of procedure per initial evaluator (hospitalist vs. cardiologist). Chi-Square test/Fisher's exact test.

Variable	Initial evaluator		Total, *n* (%)	*p*
	Hospitalists, *n* (%)	Cardiologists, *n* (%)		
Carotid Angioplasty	2 (1.8)	2 (1.2)	4 (1.4)	0.652^a^
Carotid Endarterectomy	20 (17.9)	13 (7.7)	33 (11.7)	**0.01**^b^
Aortic Aneurysm Open Repair (all types)	18 (16.1)	28 (16.6)	46 (16.4)	0.912^b^
Endovascular repair of aortic aneurysm (all types)	11 (9.8)	30 (17.8)	41 (14.6)	0.065^b^
Angioplasty for peripheral artery disease (PAD)	40 (35.7)	65 (38.5)	105 (37.4)	0.641^b^
Infrainguinal bypass graft	16 (14.3)	27 (16)	43 (15.3)	0.700^b^
Suprainguinal bypass graft	4 (3.6)	3 (1.8)	7 (2.5)	0.442^a^
Other procedures	13 (11.6)	20 (11.8)	33 (11.7)	0.954^b^
Elective surgeries	60 (51.7)	80 (48.4)	140 (50.1)	0.593^b^
Time sensitive surgeries	56 (48.3)	85 (51.6)	141 (49.9)	
Inward evaluation	65 (58)	134 (79.3)	199 (70.8)	**<0.001**^b^
Outward evaluation	47 (42)	35 (20.7)	82 (29.2)	

a Fisher's exact test.

b Chi-Square test. Bold numbers: statistically significant *p*-value.

Of the 312 arterial vascular surgeries, 124 were performed on patients evaluated by hospitalists, which included 40 angioplasties for PAD, 20 carotid endarterectomies, 18 open aortic aneurysm repairs, 16 infrainguinal bypasses, 11 endovascular aortic aneurysm repairs, 4 suprainguinal bypasses, 2 carotid angioplasties and 13 other procedures. 188 surgeries were performed on patients evaluated by cardiologists, which included 65 angioplasties for PAD, 30 endovascular aortic aneurysm repairs, 28 open aortic aneurysm repairs, 27 infrainguinal bypasses, 13 carotid endarterectomies, 3 suprainguinal bypasses, 2 carotid angioplasties, and 20 other procedures. There was a significantly higher frequency of carotid endarterectomy procedures in the hospitalists’ group, with no significant difference in the frequency of other surgeries between the groups (Table 1).

There were 140 elective surgeries (50.1%) and 141 time sensitive surgeries (49.9%). Cardiologists have evaluated 80 elective surgeries (48.4%) and 85-time sensitive surgeries (51.6%); while hospitalists have evaluated 60 elective surgeries (51.7%) and 56 time-sensitive surgeries (48.3%), with no statistical difference regarding surgeries’ nature (Table 1). The inward first preoperative evaluation was performed more frequently by cardiologists, with 134 patients in the cardiologists’ group (79.3%) versus 65 patients (58%) in the hospitalists’ group (*p <* 0.001, Table 1). None of the patients presented cardiovascular symptoms during the preoperative evaluation.

Among all 27 deaths, 7 causes were undetermined or had no autopsy, 6 patients died by postoperative infection, 3 died by AMI and 11 patients died by other causes, including arrhythmia, mesenteric ischemia, aortic prosthesis thrombosis after open abdominal aortic aneurysm repair, prostatic neoplasia and urosepsis, hypovolemic and distributive shock after open abdominal aortic aneurysm repair, suspected acute myocardial infarction (there was no myocardial necrosis markers to confirm the diagnosis) and 1 patient died during the open repair of a thoracoabdominal aortic aneurysm repair. Mortality was higher and statistically significant according to certain types of surgeries, such as open aortic aneurysm repair of all types (*n =* 12, *p <* 0.001) and angioplasty for PAD (*n =* 4, *p =* 0.011). Specifically, there were more patients submitted to open repair for infrarenal abdominal aortic aneurysm (56.3% of all cases) than justarrenal or thoracoabdominal, as shown in Table 3.

Table 2 presents hospitalization and preoperative testing data. The total length of hospital stay corresponds to the time elapsed, in days, from patient admission to hospital discharge. The average stay lasted 17.27 days in the cardiologist's group and 11.79 days in the hospitalist group (*p <* 0.001).

**Table 2:** Preoperative exams and length of hospital stay according to initial evaluator (hospitalist vs. cardiologist). Mann-Whitney test applied.

Variable	Initial evaluator	n	Mean ± Standard deviation	Median (P25‒P75)	*p*-value
Baseline creatinine levels (mg/dL)	Hospitalist	108	1.03 ± 0.81	0.9 (0.7‒1.1)	*p =* 0.788
	Cardiologist	158	1.03 ± 0.68	0.9 (0.7‒1.1)	
	Total	266	1.03 ± 0.74	1.1 (0.7‒0.9)	
Preoperative BNP levels (mg/L)	Hospitalist	10	122.29 ± 94.49	115 (41.17‒206)	*p =* 0.866
	Cardiologist	3	180.70 ± 227.11	85 (17.1‒ a)	
	Total	13	135.77 ± 126.29	85 (33.45‒224)	
Preoperative RPC levels (mg/L)	Hospitalist	49	52.64 ± 71.51	74.95 (17.7‒49)	*p =* 0.143
	Cardiologist	66	77.86 ± 114.3	26.95 (9.17‒104.52)	
	Total	115	67.11 ± 98.79	23.5 (6.8‒75.8)	
Preoperative troponin levels (pg/mL)	Hospitalist	73	16.0 ± 16.17	18 (11.5‒73)	*p =* 0.678
	Cardiologist	32	15.32 ± 14.24	12 (9‒19.05)	
	Total	105	15.79 ± 15.54	12 (8.5‒18.1)	
Total length of hospital stay (days)	Hospitalist	112	11.79 ± 12.78	7 (5‒12)	*p =***0.001**
	Cardiologist	169	17.27 ± 18.21	12 (6‒22)	
	Total	281	15.09 ± 16.46	10 (5‒20.5)	
Length of ICU stay (days)	Hospitalist	104	4.14 ± 2.8	2 (1‒4)	*p =* 0.961
	Cardiologist	159	3.46 ± 3.93	2 (1‒3)	
	Total	263	3.73 ± 3.52	2 (1‒3)	
Postoperative length of hospital stay (days)	Hospitalist	112	7.55 ± 8.95	3.5 (3‒8)	*p =* 0.164
	Cardiologist	169	9.20 ± 12.38	4 (3‒10)	
	Total	281	8.54 ± 11.15	4 (3‒9)	

a Below the detectable limit. Bold numbers: statistically significant *p*-value.

**Table 3:** Relationship between death and clinical-demographic characteristics. Fisher's exact test/Chi-Square test.

Variable	Mortality				Total	p
	Nonter		Yes			
	n	(%)	n	(%)		
**Initial evaluator**						0.609^b^
Hospitalist	100	(89.3)	12	(10.7)	112	
Cardiologist	154	(91.1)	15	(8.9)	169	
**Aortic aneurysm (all types) open repair**	34	(73.9)	12	(26.1)	46	**<0.001**^a^
**Infrarrenal**	20	(76.9)	6	(23.1)	26	**0.027**^a^
**Justarrenal**	10	(71.4)	4	(28.6)	14	**0.035**^a^
**Thoracoabdominal**	3	(60)	2	(40)	5	0.074^a^
**Endovascular repair of aortic aneurysm (all types)**	39	(95.1)	2	(4.9)	41	0.392^a^
**Carotid Endarterectomy**	32	(97)	1	(3)	33	0.222^a^
**Infrainguinal Bypass Graft**	38	(88.4)	5	(11.6)	43	0.580^a^
**Suprainguinal Bypass Graft**	6	(85.7)	1	(14.3)	7	0.511^a^
**Angioplasty for PAD**	101	(96.2)	4	(3.8)	105	**0.011**^b^
**Other surgeries**	26	(78.8)	7	(21.2)	33	0.026^a^
**Sex**						0.525^b^
Male	173	(89.6)	20	(10.4)	193	
Female	81	(92)	7	(8)	88	
**Postoperative MINS**						0.567^a^
No	94	(90.4)	10	(9.6)	104	
Yes	39	(86.7)	6	(13.3)	45	

a Fisher's exact test.

b Chi-Square test. Bold numbers: statistically significant *p*-value.

**Table 4:** Relationship between postoperative major adverse cardiovascular events (MACE) and clinical-demographic characteristics. Chi-Square test applied.

Variable	Postoperative MACE				Total	p
	No		Yes			
	n	(%)	n	(%)		
**Initial evaluator**						0.209
Hospitalist	101	(93.5)	7	(6.5)	108	
Cardiologist	146	(89)	18	(11)	164	
**Sex**						0.799
Male	174	(91.1)	17	(8.9)	191	
Female	73	(90.1)	8	(9.9)	81	
**Postoperative MINS**						0.143
No	88	(84.6)	16	(15.4)	104	
Yes	42	(93.3)	3	(6.7)	45	

The postoperative length of hospital stay (from the end of surgery to hospital discharge) and the duration of ICU stay (total number of days the patient was hospitalized in the ICU during their hospital stay) were similar between the groups. Additionally, preoperative levels of troponin, BNP, and CRP were also similar between the hospitalist and cardiologists’ groups.

There was a statistically significant difference in the stratification of cardiovascular risk in the preoperative period between the groups (*p <* 0.001). Hospitalists classified 60.7% of patients as low risk, 19.1% as intermediate risk, and 20.2% as high risk, whereas cardiologists classified 42.1% of patients as intermediate risk, 36.5% as low risk, and 21.4% as high risk.

**Figure 1:**
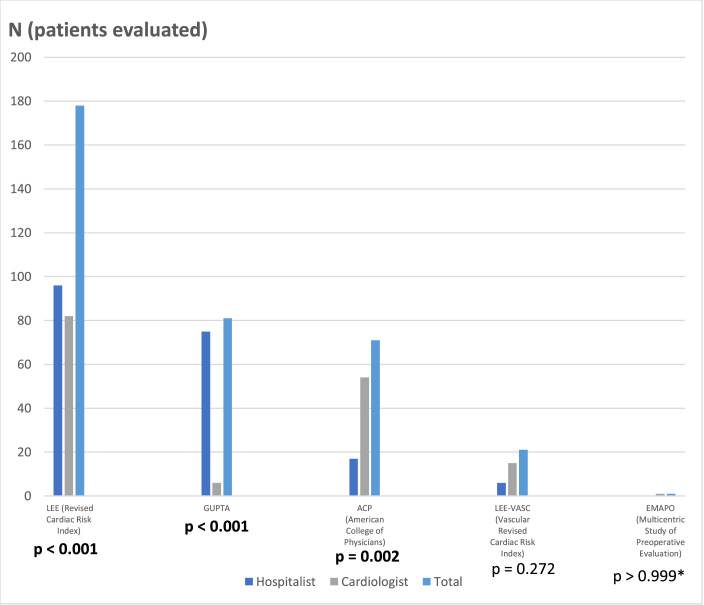
Cardiac risk scores’ choice according to initial evaluator (hospitalist vs. cardiologist). Fisher's exact test/Chi-Square test. * Fisher's exact test. # Chi-Square test. Bold numbers: statistically significant *p*-value. The sum of evaluations is greater than the number of patients studied because, in some situations, more than one score was used for the same patient.

Figure 1 shows the number of patients evaluated by each team according to the cardiac risk score used. Note that frequently more than one score was used per patient. The scores most commonly used by hospitalists were Lee and Gupta, whereas the scores most commonly used by cardiologists were Lee and ACP. Most patients in both groups were classified as ASA III, but there was no statistically significant difference in ASA physical status classification, and 98% of patients underwent general anesthesia.

**Figure 2:**
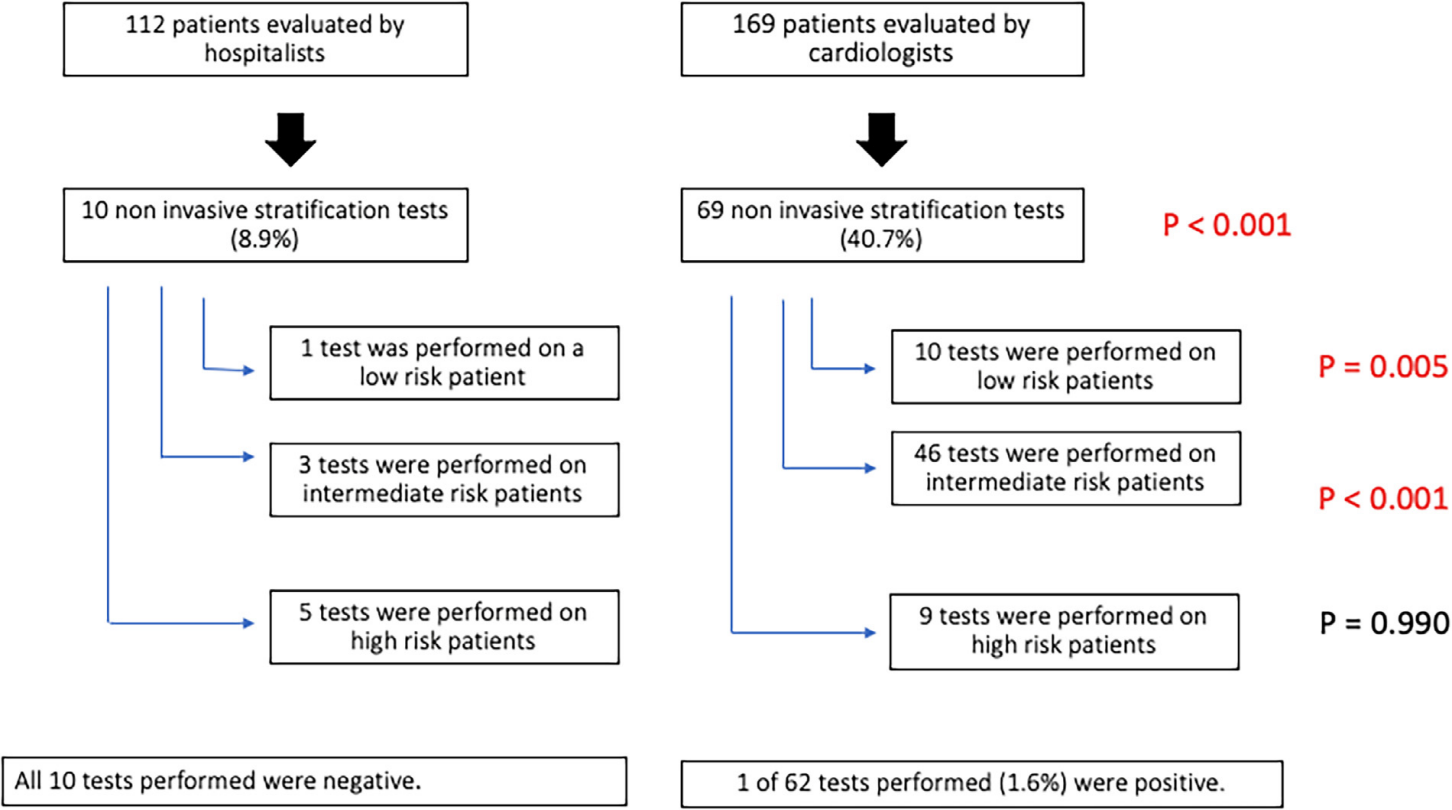
Non-invasive risk stratification tests and results according to cardiovascular risk and initial evaluator (hospitalist vs. cardiologist). Chi-Square test. Red numbers: statistically significant *p*-value. (For interpretation of the references to color in this figure legend, the reader is referred to the web version of this article.)

Of the 112 patients evaluated by a hospitalist, 10 patients were recommended for non-invasive risk stratification (8.9%), whereas 69 of the 169 patients evaluated by a cardiologist were recommended for non-invasive risk stratification (40.8%, *p <* 0.001). Figure 2 shows that cardiologists recommended significantly more non-invasive tests compared to hospitalists (*p =* 0.005) and proportionally recommended more tests when comparing the low-risk and intermediate-risk patient groups (*p <* 0.001). However, there was no statistically significant difference in the recommendation of risk stratification tests by hospitalists and cardiologists for high-risk patients (*p =* 0.99).

One of the 10 patients recommended for non-invasive risk stratification by a hospitalist did not have cardiovascular risk listed in their medical records, and all the tests performed were negative. Of the 69 patients recommended for non-invasive risk stratification by a cardiologist, 65 had documented risk stratification in the medical record and 62 underwent testing and had available reports. Among these patients, only one had a positive result and was classified as having a high cardiovascular risk (Fig. 2).

The two non-invasive risk stratification tests most commonly requested by both teams of evaluators were myocardial perfusion scintigraphy and pharmacological stress echocardiography. Both cardiologists and hospitalist groups requested one CCTA each (refer to Supplementary Table 4 in Supplementary_File).

Eleven patients were referred by a hospitalist to a cardiologist for additional evaluation, one of whom underwent urgent surgery whereas the other 10 patients were actually assessed by a cardiologist. Of these 10 patients, five were classified as high cardiovascular risk, two as intermediate risk, two as low risk, and one patient did not have cardiovascular risk listed in their medical record and underwent percutaneous revascularization.

**Figure 3:**
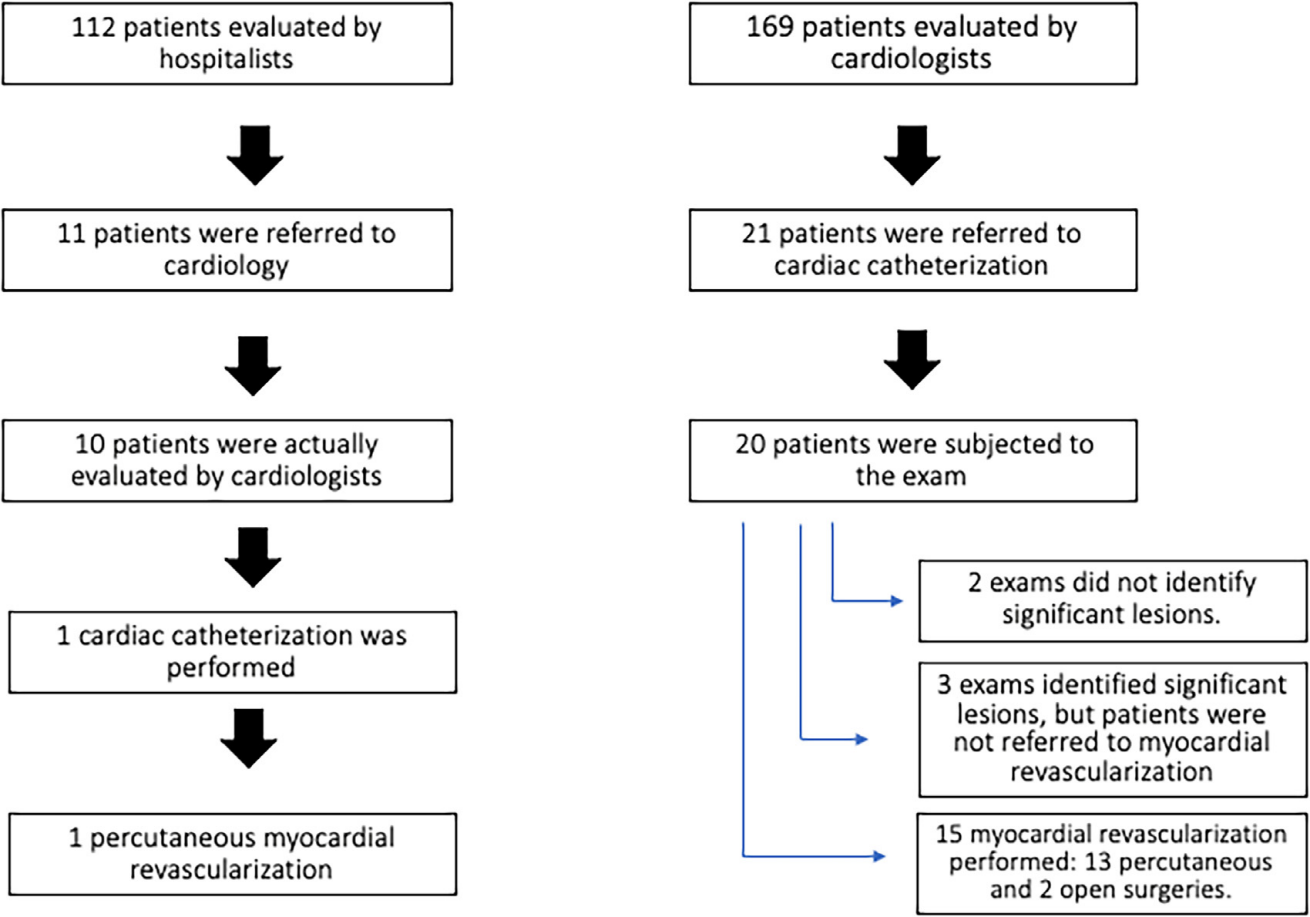
Indication for cardiac catheterization and myocardial revascularization according to initial evaluator (hospitalist vs. cardiologist).

In the cardiologists’ group, there were 21 indications for cardiac catheterization, and 20 procedures were performed as one patient required urgent surgery. Of these 20 patients, two did not have significant coronary lesions, 15 had significant abnormalities and underwent myocardial revascularization, and three had significant abnormalities but were managed with medical treatment either due to an intervention not being feasible or to high surgical risk (Fig. 3).

The average time interval between the initial preoperative evaluation (whether conducted during hospitalization or as an outpatient) and surgery was 24.63 days for patients evaluated by a hospitalist and 37.26 days for patients examined by a cardiologist (*p <* 0.001).

The average time between the initial preoperative evaluation and cardiac catheterization was 13.05 days for patients evaluated by a cardiologist and three days for those examined by a hospitalist, whereas the average time between the initial preoperative evaluation and revascularization (open or percutaneous) was eight days in the hospitalist group and 26.67 days in the cardiologists' group.

There was no statistically significant difference in the incidence of postoperative MACE when comparing the preoperative evaluation conducted by hospitalists with that conducted by cardiologists. In addition, MINS did not show a significant relationship with mortality or MACE.

Total hospitalization time, as well as Intensive Care Unit (ICU) stay, was found to be directly related to Major Adverse Cardiovascular Events (MACE). Please refer to Supplementary Tables 5, 6, 7, and 8 in Supplementary_File for the relationship between age, preoperative exams, total hospitalization time, ICU stay, mortality, and MACE.

## Discussion

Patients in the present study had similar clinical-demographic characteristics, which enabled us to compare the preoperative evaluation of arterial vascular surgeries conducted by hospitalists with those performed by cardiologists as well as the outcomes of surgery between the two groups. There was a significantly higher frequency of carotid endarterectomies in the hospitalists group, but the authors found no significant difference in the frequency of other types of surgeries between the groups (Table 1). This result is explained by the fact that this type of surgery was rarely performed during the first years of the hospital (2016‒2018), with the frequency of this type of procedure experiencing an increase from 2018, when hospitalists started being responsible for the initial preoperative cardiac evaluation.

Emergency and urgent surgeries were excluded because assessment in this context was not within the scope of this study. Additionally, when surgeries were performed in different territories (e.g., carotid and lower limb ischemia) during the same hospitalization, the authors chose not to include these types of patients in the comparative analysis. This decision was made because, aside from the small number of such patients, it would be challenging to find pairs of patients who underwent the first and second surgeries in the same arterial territories in the same sequence among the two groups (evaluated by cardiologists and hospitalists).

The definition of MACE is not strict and may vary among authors, with some also considering death resulting from these events as MACE,^1^^,^^2^ which was not considered in the present study. Mortality rates and the incidence of MACE were not significantly different between the groups. However, cardiologists requested more exams compared to hospitalists, which prolonged hospitalization stays. Cardiologists also performed first preoperative evaluation more frequently on inward patients when compared to hospitalists (79.3% vs. 58%). Due to the high demand and limited capacity to provide these tests on an outpatient basis through the Unified Health System (SUS), patients often need to be admitted to prioritize their evaluation, which leads to increased hospitalization time, bed occupancy rates, and healthcare expenditure. The authors did not find any studies regarding preoperative evaluation for any kind of elective surgeries that support this perception, and the authors believe that this work is a pioneer in this regard.

The number of postoperative hospitalization days was similar between groups. Thus, the longer total hospitalization time in the cardiologists’ group (probably as a result of more tests being requested) did not translate into shorter postoperative stays, indicating that there was no measurable benefit from the extensive evaluation done by cardiologists that could, for instance, prevent further complications.

Total hospitalization time, ICU stay, and postoperative time are expected to be longer in patients who develop MACE in the postoperative period compared to patients without MACE, as conditions such as Myocardial Infarction (MI), stroke, and arrhythmias require additional care. Alternatively, the duration of hospitalization, ICU stay, and postoperative time may not necessarily be longer in patients who die, especially if the death occurs intraoperatively or early in the perioperative period.

The results also show that the time between the initial evaluation and surgery was longer in the cardiologist group (mean of 37.26 days) compared to the hospitalist group (mean of 24.63 days), suggesting that the request for more preoperative exams and procedures by cardiologists extended the total hospitalization period.

The higher mortality found in this study is mainly due to open aortic aneurysm repairs and to peripheral artery revascularizations. According to data from DATASUS, the IT department of the Unified Health System (UHS) that collects, processes and disseminates public health information in Brazil, the mortality rate from elective open correction of abdominal aortic aneurysms operated in services of UHS (Brazil) between 2008 and 2019 is 18.6%, and 1.2% for peripheral extremity angioplasty,^13^^,^^14^ The mortality rate found in the present study is higher (23.1% and 3.8%, respectively), and it should be noted that the data periods from the UHS (2008–2019) and those from our study (2016–2020) are not exactly the same, but there is some overlap.

Other aortic aneurysm topography repairs (justarrenal, thoraco-abdominal), and open bypass grafts (suprainguinal and infrainguinal) were relatively rare in the sample of this study, hindering more robust statistical comparisons. The authors believe that the high mortality found in our study is due to the relatively low volume of these surgeries performed in our center. Other studies have shown that services with low volumes of lower limb revascularizations and open repair of abdominal aortic aneurysms (less than 20 cases per year) present higher mortality and more postoperative complications than centers with higher volumes.^15^^,^^16^

Our findings indicate that hospitalists requested fewer exams to perform a preoperative assessment, which reduced the length of hospital stay without changing the outcome (MACE and mortality rates). The importance of the hospitalists is recognized in other studies in the preoperative period,^9^^,^^10^ but it's also well established in the postoperative period, with studies showing lower postoperative complications, mortality, and hospital costs when a hospitalist monitors patients post-operatively together with the surgical team.^17^^,^^18^

Previous studies have shown that more invasive approaches in preoperative evaluation do not always reduce morbidity and mortality or guarantee better perioperative outcomes.^19^ For instance, it is known that preventive cardiac revascularization before arterial surgery in patients with stable coronary artery disease does not impact short- and long-term postoperative mortality rates.^20^, ^21^, ^22^ Furthermore, consistent with the present findings, some studies have shown that there is no benefit in performing non-invasive risk stratification in low- or intermediate-risk patients, which may be of low predictive value for cardiac events and lead to possible delays in performing vascular surgeries.^22^, ^23^, ^24^, ^25^, ^26^ Thus, it is important that physicians conduct preoperative cardiac evaluations conscientiously, weighing the risks and benefits of each intervention, with precise indications for tests, avoiding surgery delays and overspending already scarce resources.

## Limitations

Due to the retrospective nature of the study, the data are extracted from medical records, which can sometimes provide incomplete information. Some of the non-invasive risk stratification tests requested for patients in the cardiologist's group either had missing information on results or were not performed (10.14%), and some patients included in the study did not have their preoperative risk conclusion described in the medical records (9.97%). Additionally, the number of patients analyzed was determined by convenience (patients operated over a five-year period), without previous calculation of the sample's statistical power, which is a potential limitation for the interpretation of the present results. Furthermore, all limb revascularizations were classified as time-sensitive, but there is a possibility that a small proportion of these patients was not operated on for critical limb ischemia, but for limiting claudication, a condition that, for itself, is not time-sensitive. It is not routine in this service to revascularize limiting claudication patients, but few patients might have been submitted to surgery due to this condition. Other significant data not collected in this study is the functional status of the patients, which influences the appropriate indication of complementary cardiologic evaluation, and the authors recognize it as a limitation (although we believe that the proportion of patients with different functional statuses should not be significantly different between those evaluated by cardiologists and those evaluated by hospitalists).

## Conclusion

No significant differences were found in the incidence of adverse outcomes among patients undergoing arterial vascular surgery when comparing preoperative evaluations conducted by cardiologists and hospitalists. The larger number of tests requested, and the higher frequency of cardiac revascularization procedures did not have any significant positive impact on mortality and MACE but further delayed surgeries instead, increasing the demand for financial and human resources. Healthcare managers ‒ not only, but especially in countries with low per capita income ‒ should consider the findings of this study and ensure that physicians make appropriate use of resources in the preoperative assessment of arterial vascular surgeries by critically reflecting with the help of the best scientific evidence available. Preoperative assessments by hospitalists were associated with similar clinical outcomes but with less use of hospital resources and reduced hospital stay.

## Authors’ contributions

A.S.M. was involved in all stages of the study, from methodology, data curation and formal analysis, to writing the original draft, review and editing. L.T.C. and I.C.M. assisted in the writing of the original draft. V.D., L.O.V., and F.S.O.P. assisted in data curation. N.W. and M.T. supervised the entire study, from project administration, data curation and formal analysis to article writing (original draft, review and editing).

As an observational study, this manuscript is in accordance with the STROBE (Strengthening the Reporting of Observational Studies in Epidemiology) statement.

## Funding

The research did not receive any specific grant from funding agencies in the public, commercial, or not-for-profit sectors.

## Declaration of competing interest

The authors declare no conflicts of interest.

## Appendix Supplementary materials

Image, application 1

Image, application 2
