# Supplementary material for: Preoperative evaluation profile of patients undergoing arterial vascular surgery in a tertiary hospital
Source: Clinics (Sao Paulo). 2024 Jul 25;79:100445. doi: 10.1016/j.clinsp.2024.100445 (PMC11338055; doi:10.1016/j.clinsp.2024.100445)
Supplement: Supplementary file 1 [file mmc1.docx]

**CLINICS-D-23-00774_ Supplementary Material**

**Supplementary Table 1** Clinical-demographic characteristics of patients.

| Initial evaluator | | |  |  |
| --- | --- | --- | --- | --- |
| General practitioner n (%) | | Cardiologist n (%) | Total n (%) | **p** |
| Sex | | | | 0.613 |
| Male | 75 (67.0) | 118 (69.8) | 193 (68.7) |  |
| Female | 37 (33.0) | 51 (30.2) | 88 (31.3) |  |
| SAY | 97 (86.6) | 144 (85.2) | 241(85.8) | 0.742 |
| Previous stroke | 27 (24.1) | 39 (23.1) | 66 (23.5) | 0.842 |
| Diabetes Mellitus (with or without insulin therapy) | 53 (47.3) | 81 (47.9) | 134 (47.7) | 0.920 |
| Diabetes Mellitus, on insulin therapy | 19 (17.0) | 39 (23.1) | 58 (20.6) | 0.215 |
| Chronic Kidney Disease | 13 (11.6) | 29 (17.2) | 42 (14.9) | 0.201 |
| Smoking |  |  |  | 0.271 |
| Previous | 48 (45.7) | 77 (44.5) | 117 (44.0) |  |
| Current | 33 (31.4) | 61 (39.4) | 94 (36.2) |  |
| Never | 24 (22.9) | 25 (16.1) | 49 (18.8) |  |
| History of myocardial revascularization | 16 (14.3) | 24 (14.2) | 40 (14.2) | 0.984 |
| Open myocardial revascularization | 7 (6.3) | 10 (5.9) | 17 (6) | 0.909 |
| Percutaneous coronary intervention | 8 (7.1) | 14 (8.3) | 22 (7.8) | 0.727 |
| Total | 112 (100.0) | 169 (100.0) | 281 (100.0) |  |
| Chi-square test; | | | |  |

SAY**,** Systemic Arterial Hypertension.

**Supplementary Table 2** Medication used in the preoperative period.

| Initial evaluator | | |  |  |
| --- | --- | --- | --- | --- |
| General practitioner n (%) | | Cardiologist n (%) | Total n (%) | **p** |
| Beta Blocker use | 43 (38.4) | 66 (39.1) | 109 (38.8) | 0,911 |
| Aspirin intake |  |  |  | 0.424# |
| No, due to allergy/intolerance | 1 (0.9) | 0 (0) | 1(0.4) |  |
| No, but it was initiated | 12 (10.7) | 13 (7.7) | 25 (8.9) |  |
| Yes | 95 (84.8) | 148 (87.6) | 243 (86.5) |  |
| No, and it was chosen not to initiate | 4 (3.6) | 8 (4.7) | 12 (4.3) |  |
| Statin use | 110 (98.2) | 160 (94.7) | 270 (96.1) | 0.209 |
| Clopidogrel use |  |  |  | 0.759# |
| No, due to allergy/intolerance | 1 (0.9) | 1 (0.5) | 2 (0.7) |  |
| No, but it was initiated | 36 (32.1) | 62 (37.9) | 98 (34.9) |  |
| Yes, and it was suspended | 6 (5.4) | 12 (6.6) | 18 (6.4) |  |
| Yes, and it was not suspended | 4 (3.6) | 3 (1.6) | 7 (2.5) |  |
| No, and it was not initiated | 65 (58.0) | 91 (53.3) | 156 (55.5) |  |
| Total | 112 (100.0) | 169 (100.0) | 281 (100.0) |  |
| Chi-square test; # Likelihood ratio test | | | |  |

**Supplementary Table 3** Age.

| Variable | Initial evaluator | Mean | SD | Median | N | p |
| --- | --- | --- | --- | --- | --- | --- |
| Age (years) | General practitioner | 66.07 | 10.97 | 68 | 112 | 0.971* |
|  | Cardiologist | 66.12 | 10.18 | 67 | 169 |  |
|  | Total | 66.10 | 10.49 | 68 | 281 |  |

**Supplementary Table 4** Relationship between indication and type of non-invasive diagnostic tests requested for risk stratification and positivity rate.

| Initial evaluator | | |  |  |
| --- | --- | --- | --- | --- |
| General practitioner n (%) | | Cardiologist n (%) | Total n (%) | **p** |
| Non-invasive diagnostic tests for risk stratification | | | | **<0.001** |
| No | 102 (91.1) | 100 (59.2) | 202 (71.9) |  |
| Yes | 10 (8.9) | 69 (40.8) | 79 (28.1) |  |
| Myocardial perfusion scintigraphy | | | | **0.001** |
| No | 110 (98.2) | 145 (85.8) | 255 (90.7) |  |
| Yes | 2 (1.8) | 24 (14.2) | 26 (9.3) |  |
| Coronary CT angiography | | | | >0.999* |
| No | 111 (99.1) | 168 (99.4) | 279 (99) |  |
| Yes | 1 (0.9) | 1 (0.6) | 2 (0.7) |  |
| Pharmacological stress echocardiogram | | | | **<0.001** |
| No | 105 (93.8) | 122 (72.2) | 227 (100) |  |
| Yes | 7 (6.3) | 47 (27.8) | 54 (19.2) |  |
| Total | 112 (100) | 182 (100) | 294 (100) |  |
| Did the risk stratification test come back positive? | | | | >0.999* |
| No | 10 (100) | 61 (98.4) | 71(98.6) |  |
| Yes | 0 | 1(1.6) | 1(1.4) |  |
| Total | 10 (100) | 62 (100) | 76 (100) |  |
| Chi-square test; * Fisher's exact test | | | |  |

**Supplementary Table 5** Relationship between age and mortality.

| Variable | Death | Mean | SD | Min. | | Max. | N | P |
| --- | --- | --- | --- | --- | --- | --- | --- | --- |
| Age (years) | No | 66.23 | 9.93 | 27 | 85 | | 254 | 0.516 |
|  | Yes | 64.85 | 14.95 | 20 | 79 | | 27 |  |
|  | Total | 66.10 | 10.49 | 20 | 85 | | 281 |  |
| T-Student test | | | | | | | | |

**Supplementary Table 6** Relationship between preoperative tests, ICU length of stay, and mortality.

| Variable | Death | Mean | P25 | Median | P75 | Min. | Max. | N | P |
| --- | --- | --- | --- | --- | --- | --- | --- | --- | --- |
| Preoperative BNP (pg/mL) | No | 101.20 | 17.77 | 80.5 | 162.5 | 10 | 261 | 10 | 0.176* |
|  | No | 251.00 | 53 | 260 | - | 53 | 440 | 3 |  |
|  | No | 135.77 | 33.45 | 85 | 224 | 10 | 440 | 13 |  |
| Preoperative CRP (pg/mL) | No | 57.20 | 5.9 | 22.9 | 61 | 0.3 | 290 | 103 | **0.027*** |
|  | Yes | 152.23 | 16.05 | 99.65 | 184.3 | 10.4 | 640 | 12 |  |
|  | Total | 67.11 | 6.8 | 23.5 | 75.8 | 0.3 | 640 | 115 |  |
| Troponin (pg/mL) | No | 14.08 | 8 | 12 | 17.5 | 0 | 61 | 97 | **0.024*** |
|  | Yes | 36.56 | 13.12 | 22 | 61.9 | 5 | 114 | 8 |  |
|  | Total | 15.79 | 8.5 | 12 | 18.1 | 5 | 114 | 105 |  |
| ICU Length of Stay (days) | No | 2.63 | 1 | 2 | 3 | 0 | 23 | 238 | 0.165* |
|  | Yes | 4.25 | 1 | 2.5 | 6.25 | 0 | 21 | 24 |  |
|  | Total | 2.78 | 1 | 2 | 3 | 0 | 23 | 262 |  |
| Baseline creatinine (mg/dL) | No | 1.02 | 0.7 | 0.9 | 1.1 | 0.3 | 8.5 | 241 | 0.103* |
|  | Yes | 1.13 | 0.7 | 1.26 | 1.6 | 0.3 | 1.8 | 25 |  |
|  | Total | 1.03 | 0.7 | 0.9 | 1.1 | 0.3 | 8.5 | 266 |  |
| * Mann-Whitney test; - below detectable levels | | | | | | | | | |

**Supplementary Table 7** Relationship between age and MACE.

| Variable | MACE | Mean | SD | Min. | Max. | N | p |
| --- | --- | --- | --- | --- | --- | --- | --- |
| Age (years) | No | 65.86 | 10.80 | 20 | 85 | 247 | 0.197 |
|  | Yes | 68.72 | 7.04 | 53 | 79 | 25 |  |
|  | Total | 66.13 | 10.53 | 20 | 85 | 272 |  |
| T-Student test | | | | | | | |
