# Supplementary material for: Preoperative evaluation profile of patients undergoing arterial vascular surgery in a tertiary hospital
Source: Clinics (Sao Paulo). 2024 Jul 25;79:100445. doi: 10.1016/j.clinsp.2024.100445 (PMC11338055; doi:10.1016/j.clinsp.2024.100445)
Supplement: Supplementary file 2 [file mmc2.docx]

**Supplementary Table 8: Relationship between preoperative tests, total length of hospital stay, ICU length of stay, and postoperative MACE**

| Variable | MACE | Mean | P25 | Median | P75 | Min. | Max. | N | p |
| --- | --- | --- | --- | --- | --- | --- | --- | --- | --- |
| Preoperative BNP  (pg/mL) | No | 110.42 | 25.72 | 80.5 | 179.5 | 10 | 261 | 12 | 0.108* |
|  | Yes | 440.00 | 440 | 440 | 440 | 440 | 440 | 1 |  |
|  | Total | 135.77 | 33.45 | 85 | 224 | 10 | 440 | 13 |  |
| Preoperative CRP  (pg/mL) | No | 62.48 | 6.1 | 23.5 | 74.1 | 0.3 | 640 | 103 | 0.163* |
|  | Yes | 106.84 | 12.72 | 39.25 | 213.55 | 5 | 392.9 | 12 |  |
|  | Total | 67.11 | 6.8 | 23.5 | 75.8 | 0.3 | 640 | 115 |  |
| Troponin (pg/mL) | No | 13.57 | 8 | 12 | 15.7 | 0 | 73.8 | 97 | **<0.001*** |
|  | Yes | 42.70 | 18.37 | 32 | 57.57 | 17 | 114 | 8 |  |
|  | Total | 15.79 | 8.5 | 12 | 18.1 | 0 | 114 | 105 |  |
| Total Length of Hospital Stay (days) | No | 13.59 | 5 | 9 | 17 | 2 | 118 | 247 | **<0.001*** |
|  | Yes | 32 | 18 | 26 | 36.5 | 5 | 128 | 25 |  |
|  | Total | 15.28 | 5 | 10 | 20.5 | 2 | 128 | 272 |  |
| ICU Length of Stay (days) | No | 2.42 | 1 | 2 | 3 | 0 | 22 | 232 | **<0.001*** |
|  | Yes | 6.87 | 2 | 5 | 8 | 1 | 23 | 23 |  |
|  | Total | 2.82 | 1 | 2 | 3 | 0 | 23 | 255 |  |
| Baseline creatinine (mg/dL) | No | 1.01 | 0.7 | 0.9 | 1.1 | 0.3 | 8.5 | 242 | 0.295* |
|  | Yes | 1.20 | 0.7 | 0.9 | 1.57 | 0.3 | 3.8 | 24 |  |
|  | Total | 1.03 | 0.7 | 0.9 | 1.1 | 0.3 | 8.5 | 266 |  |
| * Mann-Whitney test | | | | | | | | | |
